# Nano Matrix Soft Confectionary for Oral Supplementation of Vitamin D: Stability and Sensory Analysis

**DOI:** 10.3390/gels8050250

**Published:** 2022-04-19

**Authors:** Mohammad Zubair Ahmed, Anshul Gupta, Musarrat Husain Warsi, Ahmed M. Abdelhaleem Ali, Nazeer Hasan, Farhan J. Ahmad, Ameeduzzafar Zafar, Gaurav K. Jain

**Affiliations:** 1 Department of Pharmaceutics, School of Pharmaceutical Education and Research, Jamia Hamdard, New Delhi 110062, India; zubair_amgaz@hotmail.com (M.Z.A.); nazeerhasan1994@gmail.com (N.H.); farhanja_2000@yahoo.com (F.J.A.); 2 Department of Pharmaceutics, Delhi Pharmaceutical Sciences and Research University, New Delhi 110017, India; gupta.ansh.198@gmail.com; 3 Department of Pharmaceutics and Industrial Pharmacy, College of Pharmacy, Taif University, P.O. Box 11099, Taif 21944, Saudi Arabia; a.mali@tu.edu.sa; 4 Department of Pharmaceutics, College of Pharmacy, Jouf University, Sakaka 72341, Al-Jouf, Saudi Arabia; azafar@ju.edu.sa; 5 Center for Advanced Formulation Technology, Delhi Pharmaceutical Sciences and Research University, New Delhi 110017, India

**Keywords:** vitamin D, nanoemulsion, gelled matrices, texture analysis, gelatin, sensory evaluation, gummy

## Abstract

Vitamin D deficiency distresses nearly 50% of the population globally and multiple studies have highlighted the association of Vitamin D with a number of clinical manifestations, including musculoskeletal, cardiovascular, cerebrovascular, and neurological disorders. In the current study, vitamin D oil-in-water (O/W) nanoemulsions were developed and incorporated in edible gummies to enhance bioavailability, stability, and patient compliance. The spontaneous emulsification method was employed to produce a nano-emulsion using corn oil with tween 20 and lecithin as emulsifiers. Optimization was carried out using pseudo-ternary phase diagrams and the average particle size and polydispersity index (PDI) of the optimized nanoemulsion were found to be 118.6 ± 4.3 nm and 0.11 ± 0.30, respectively. HPLC stability analysis demonstrated that the nano-emulsion prevented the degradation and it retained more than 97% of active vitamin D over 15 days compared to 94.5% in oil solution. Similar results were obtained over further storage analysis. Vitamin D gummies based on emulsion-based gelled matrices were then developed using gelatin as hydrocolloid and varying quantities of corn oil. Texture analysis revealed that gummies formulated with 10% corn oil had the optimum hardness of 3095.6 ± 201.7 g on the first day which remained consistent on day 45 with similar values of 3594.4 ± 210.6 g. Sensory evaluation by 19 judges using the nine-point hedonic scale highlighted that the taste and overall acceptance of formulated gummies did not change significantly (*p* > 0.05) over 45 days storage. This study suggested that nanoemulsions consistently prevent the environmental degradation of vitamin D, already known to offer protection in GI by providing sustained intestinal release and enhancing overall bioavailability. Soft chewable matrices were easy to chew and swallow, and they provided greater patient compliance.

## 1. Introduction

Vitamin D or calciferol is a lipid-soluble vitamin which is a combination of various steroidal derivatives. These derivatives include: cholecalciferol (vitamin D3), which is a derivative of cholesterol, calcidiol (partially active hydroxylated form of cholecalciferol), calcitriol (dihydroxylated active form), ergocalciferol (D2), and its mono and dihydroxylated derivatives [1]. Vitamin D is both endogenous and exogenous. The human skin epidermis, when exposed to ultraviolet-B (UVB) radiation present in sunlight, produces vitamin D3 from 7-dehydrocholesterol endogenously. Exogenous vitamin D is sourced from dietary food, such as dairy, oily fish, liver, egg yolk, etc., and supplements. Since the natural food options which can provide exogenous vitamin D are quite limited, supplementation is increasingly encouraged to counter its deficiency. Vitamin D in general, refers to vitamin D3. Its primary circulating form 25-hydroxyvitamin D is converted to active metabolite 1,25-dihydroxyvitamin D within the human body [2].

Individuals having serum vitamin D levels (i.e., 25-hydroxyvitamin D [25(OH)D]) below 20 ng per milliliter or 50 nmol per liter (deemed appropriate level by the Institute of Medicine in 2011) are considered vitamin D deficient. In 2011, after a comprehensive analysis of the literature, the Institute of Medicine (IOM) reached the conclusion that a 25(OH)D concentration of 20 ng/mL or above in blood was adequate for optimal bone health [3,4,5]. Vitamin D deficiency is associated with a number of clinical manifestations in people of different age groups. It is predominantly associated with bone health, and vitamin D deficiency is well understood to contribute to musculoskeletal disorders. Without vitamin D, just 10–15% of dietary calcium and about 60% of phosphorus are absorbed. [2]. Vitamin D deficiency results in musculoskeletal fatigue, and low vitamin D levels have been observed in conjunction with rheumatoid arthritis, multiple sclerosis, and arthritis, closely associated with many cardiovascular risk factors, e.g., myocardial heart infarction, congestive heart failure [6], diabetic cardiovascular disease, and peripheral arterial disease. A number of studies have suggested the role of vitamin D deficiency in increased cerebrovascular accident (CVA) risk and it has been linked to an elevated risk of depression and schizophrenia [7,8,9]. Vitamin D modulates the role of B and T-lymphocytes and regulates cell differentiation and proliferation [10,11]. Multiple studies have acknowledged that women represent a high risk population for vitamin D deficiency, with pregnant women being at higher risks, leading to multiple pregnancy complications, e.g., gestational diabetes and preeclampsia [12]. Vitamin D deficiency is prevalent across the world, with an estimated 1 billion people suffering from the condition [13]. According to several reports, 40–100% of elderly people in the U.S. and European countries are vitamin D deficient. Furthermore, various studies have shown low vitamin D status regardless of age, gender, and geography [2,13,14,15]. According to a meta-analysis report from 2015, vitamin D deficiency was 35% more frequent among obese people irrespective of age or demography [16]. Vitamin D deficiency has been associated with populations having higher levels of melanin in their skin and the populations that use extensive skin care, notably in Middle East nations [13,17].

The Institute of Medicine (IOM) has published guidelines for vitamin D supplementation, suggesting 600 IU/day vitamin D for most of the population aging between one and 70 years. The Recommended Dietary Allowance (RDA) of vitamin D for individuals above 70 years of age is 800 IU per day and for infants up to one year of age is 400 IU a day [18]. For the treatment and prevention of vitamin D deficiency, the Endocrine Society in the United States recommended more than 30 ng/mL of serum 25(OH)D concentrations to be achieved, more preferably in the range of 40–60 ng/mL [4]. To reach a serum 25(OH)D level of 30 ng/mL, all deficient adults should be provided with either 50,000 IU weekly of vitamin D3 or 6000 IU daily for 8 weeks. A daily maintenance dose of 1000 IU in patients up to 18 years and 1500–2000 IU daily in patients aging 18–50 years is recommended by The Endocrine Society [4,19].

Limitations with supplemental solid oral delivery: Oral delivery is perceived as most reliable route for the administration of active pharmaceutical ingredients (APIs). More than 60% of all medicines comprise of solid oral dosage forms due to ease of manufacture, availability of large number of excipients, cheaper development process, more accurate dosing compared to oral liquids, and ease of handling and dispensing [20]. Since it is non-invasive, patients are more comfortable compared to other routes. Major disadvantages with oral formulations include low gastric and intestinal solubility, which reduces the bioavailability of large number of pharmaceuticals and rapid stomach breakdown, possibly followed by degradation of the API by gastrointestinal (GI) tract enzymes [21]. Another critical concern is patient compliance among children and the elderly majorly due to dysphagia. A questionnaire research revealed that 26% of 6158 patients reported difficulties swallowing tablets [22]. Another study found that 37.4% of all participants (*n* = 1051) reported having trouble swallowing tablets and capsules [17]. When it comes to supplementation (either prophylactic or treatment), where a medication or dose is not usually regarded equally relevant or critical as other urgent needs or treatment medications, the graph of compliance further falls. Adults with dysphagia or those reluctant to take medications usually ignore the supplementary pills. Parents usually miss the supplements when given as solid orals due to reluctance of children to take the pills.

Limitations with vitamin D stability and bioavailability: Cholecalciferol is highly susceptible to environmental degradation (sunlight, heat, and oxygen), and readily undergoes isomerization or oxidation when exposed to open air in its raw form, resulting in a loss of its functioning and physiological implications. Several studies report that it is poorly water-soluble and solubilizing vitamin D in some carrier oils protects it from the effects of heat or oxidation [23,24,25]. Vitamin D has very limited bioavailability, which limits its effectiveness when given as an oral supplement, and higher doses are required to maintain effective levels. It has been observed that only around half (~50%) of the Vitamin D3 consumed orally is absorbed [26].

Lipid-based formulations have gained commercial recognition by improving the oral bioavailability and solubility of poor water-soluble drugs. They enhance the drug absorption by solubilizing the poorly water soluble or insoluble drugs and promoting the emulsification process in the GIT. The application of nano-emulsions (NEs) in the food sector has attracted growing interest owing to the changes in the physicochemical attributes and biological efficiency associated with particle size modifications. Particle size reduction in emulsion-based formulations can have a variety of effects that may benefit various food and supplemental applications: (i) enhanced stability against aggregation and separation of droplets under gravity; (ii) greater visual clarity; and (iii) improved oral bioavailability [27,28]. In one of the studies conducted by Salvia-Trujillo et al., lipid droplets of a relatively smaller size were digested considerably quicker in simulated GI fluid suggesting mixed micelles that solubilize lipophilic vitamins may develop more quickly in the small intestine [27]. Because of their potential to yield robust and transparent delivery systems with excellent oral bioavailability, NEs are particularly well suited for encapsulating lipophilic nutraceuticals [29]. NEs are dual phased thermodynamically stable systems comprising of at least two immiscible liquids having droplet sizes in the nanometer range usually below 200 nm. They have good kinetic stability upon storage and can be fabricated with minimal surfactant concentrations [30,31]. High-energy emulsification technologies, such as using ultrasonic generators or high-pressure homogenizers, can be used to synthesize NEs. Nano emulsion-based soft chewable matrices that are easy to chew and swallow and have the potential to entrap the medication, giving sustained, controlled release while avoiding quick disintegration in the stomach, have shown promise as delivery systems.

Vitamins in chewable gummy form have become increasingly common today among both the young and adults. Gummy supplements are reported to accommodate the ‘pill fatigue’ of consumers and to mask the ‘vitamin taste’ that is often very poor or has an odious smell [32]. Nowadays, children with complete dentition readily embrace gelled gummies and candies because of their good taste and chewability, since they are frequently flavored with various fruity and other lucrative flavors and extracts and are sweet in nature. Presently, two out of the top 10 vitamin D supplements are gummies. This is due to the widespread acceptability of gummy soft chew supplements among people of all age groups. It has also been evident in many studies that children’s nutritional status has been improved with chewable vitamins [32,33]. In the light of above considerations, a nano-emulsion-based soft confectionary system was developed, having a soft chewable matrix, which was followed by an evaluation of its texture profile, dissolution, and consumer compliance.

## 2. Materials

Vitamin D (97–99% pure) was procured from HiMedia Laboratories Pvt. Ltd. Mumbai, India. Cold pressed pure corn oil was purchased from Deve herbes, Delhi, India. Gelatin 160 g bloom strength, Poloxamer 188, lecithin, and sucralose were purchased from Sigma-Aldrich, Inc., St. Louis, MO, USA. Transcutol and Poloxamer 407 were purchased from Gattefosse, Saint-Priest, France. Tween 20, Tween 80, Tween 60, and Span 20 were bought from Loba Chemie Pvt. Ltd., Mumbai, India. HPLC grade methanol and water were bought from Merck, Mumbai, India. Food Safety and Standards Authority of India (FSSAI) grade sorbitol/xylitol, sucralose, citric acid, and flavors were purchased from local vendors.

## 3. Experimental Design

### 3.1. Preparation and Optimization of Vitamin D Nanoemulsion

The oil phase of nanoemulsion was selected based on the reported solubility of vitamin D in various oils [34,35,36,37,38,39]. Surfactant and co-surfactant were selected based on their ability to spontaneously emulsify corn oil [40]. Briefly, 100 mg of corn oil was dissolved in 3 mL of methylene chloride and then surfactant/co-surfactant (10 mL) was added and stirred at 200 rpm (at 40 °C) using magnetic stirrer to remove methylene chloride. Thereafter, 1 mL of sample was withdrawn and in it 10 mL of distilled water was added gradually, and percentage transmittance was determined at 339 nm wavelength using a UV spectrophotometer [41]. All the measurements were performed in triplicate.

Nanoemulsion optimization was performed by aqueous titration method followed by pseudo-ternary phase diagram as described previously [42,43]. Corn oil containing a weighed quantity of vitamin D was admixed with the combination of surfactant and co-surfactant (Smix) in different ratios (1:1, 2:1, 1:2, 1:3 and 3:1). The mixtures were then titrated with water, vortexed, and analyzed visually for their appearance, clarity, or turbidity (Table 1) and plotted as ternary phase diagram. Clear dispersions (nanoemulsions) with a minimum concentration of Smix were selected for further evaluation [44]. 

### 3.2. Characterization of Vitamin D Nanoemulsion 

The average particle size of the fabricated nanoemulsion was evaluated using Zetasizer Nano ZS90 (Malvern Instruments, Malvern, UK) via dynamic light scattering (DLS). An ambient temperature of 25 °C and scatter angle of 173° were set to carry out the measurements. To eliminate the overlapping effects of scattering, a dilution of 500 times was performed with buffer solution of equivalent pH before measurements. A disposable capillary cell of 1 mL was employed for zeta potential measurement.

To analyze morphology, a Morgagni 268D transmission electron microscope (TEM, Fei Electron Optics, Eindhoven, The Netherlands) functioning at 80 kV was employed. On a 400-mesh copper panel coated with carbon foil, one drop of each sample previously diluted 100 times was applied and allowed to dry for two minutes. A negative stain of 2% phosphotungstic acid was then applied to the sample slide and allowed to dry before examination. Observations were conducted at room temperature, using a smaller objective aperture, a lower accelerating voltage of 80 kV, and at increasing magnifications [44].

### 3.3. Stability Study of Vitamin D Nanoemulsion 

The high-performance liquid chromatography (HPLC) analysis was employed for quantification of vitamin D. Waters e2695 separations Module HPLC system (Waters Co., Milford, MA, USA) equipped with autosampler, Waters 2489 dual wavelength UV detector and a column heater was used. The symmetry C8 column by Waters, having 100 Å pore size with 4.6 mm internal diameter, 5 µm particle size, and 250 mm length, was used for the stationary phase and Empower™ software (Version 3, feature release 4) from Waters was used to process and analyze the results.

A primary stock solution of 1 µg/mL was prepared by dissolving vitamin D in HPLC grade methanol. Hence, 50 mg vitamin D was serially diluted to obtain standard solution of desired concentration. The following process parameters were utilized for separation: temperature was maintained at 25 ± 2 °C, methanol and HPLC grade water in a ratio of 90:10 (*v*/*v*) were employed as mobile phase, 1.0 mL/min flow rate was set and maintained, sample cooler was set to 15 °C, and detection was carried out at 265 nm. The standard solution was analyzed 5 times (*n* = 5) for regression.

Vitamin D nanoemulsion was freshly prepared and stored in light resistant container. Since vitamin D degrades rapidly in the aqueous solution, a comparison was made with the oil solution of vitamin D prepared by mixing a weighed quantity of vitamin D in corn oil. One mL of both samples was diluted to 100 mL using HPLC grade methanol and were then analyzed on day 0, day 15 and day 30.

### 3.4. Soft Confectionary (Nano Gummy) Preparation 

**Figure 1:**
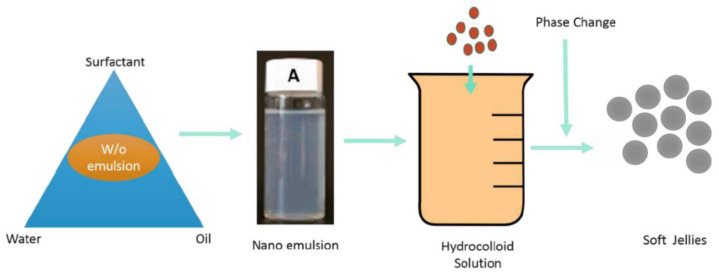
Formulation development process from nano emulsion optimization to gummy formation.

The nanoemulsion based gelled matrix system was developed using gelatin (hydrocolloids), as shown in Figure 1. The matrices were made from a blend of type A and type B gelatin with a bloom strength of 160 g. Different combinations of sweeteners (sorbitol, xylitol, sucralose), citric acid, and flavors (coffee, lime, vanilla, orange, candy) were tested. 

Briefly, gelatin types A and B in equal quantity were dissolved in a small quantity of water heated to 60 °C by stirring at 100 rpm. To this, the previously developed o/w nanoemulsion was added, and the mixture was stirred for 2 min. Sweeteners were then added followed by citric acid, flavoring agent, and corn oil (plasticizer). The influence of corn oil on the matrix properties and product acceptability was studied. The formula of the developed Nano gummy using 10% corn oil is shown in Table 2. 

### 3.5. Texture Profile Analysis of Nano Gummy

The textural properties of gummy confections were evaluated using two-bite compression tests [45]. Gummies were formulated with different corn oil concentrations (0%, 10%, 20% and 30%) and textural attributes, including hardness and stickiness, were measured. A TA.XT2i Texture Analyzer (Stable Micro Systems, Godalming, UK) was used for the evaluation. All measurements were made using a 5 kg load cell. A cylinder aluminum plate probe with a diameter of 7.5 cm, lubricated using the corn oil was lowered at a speed of 1 mm/s to compress 50% of confectionary jelly cube measuring 10 mm^3^ placed on the fixed bottom surface. The equipment was adjusted to zero, trigger force was kept at 0.05 N, and the plate was automatically lowered until the bottom surface of the plate just contacted the table. All the measurements were made in triplicate. Data were obtained and analyzed using Stable Micro System’s Exponent software, which evaluates the force–time curve formed during the compression of the sample [46,47].

### 3.6. Sensory Evaluation of Nano Gummy

Sensory assessment is accomplished by documenting the outcome of the assessor’s replies based on the parameters of taste, texture/appearance, aroma, and overall liking after a comprehensive study. A questionnaire was prepared, and sensory assessments were performed using a qualitative 9-point hedonic scale, with 1 being least likely and 9 being most. Participant were given three different gummies, viz. Placebo gummy (nano gummy without vitamin D), Nano gummy, and Marketed gummy (Azveston Healthcare Pvt. Ltd., Bengaluru, India) on Day 1 and again on Day 45 after storage. Volunteers were screened based on inclusion and exclusion criteria and were informed about the study objectives and recruited after providing informed consent. Gender-neutral healthy volunteers who spoke and understood English, aged between 18 and 45 years, and had no medical history or known sensitivities to the materials used in gummy fabrication were eligible to participate. Participants younger than 18 years old, smokers, those having flu or similar manifestation, those who did not speak or comprehend the language of choice, and those who had known sensitivities to the materials used in gummy production were all excluded. Oral hygiene product, food and aerated drinks were restricted 2-h before and throughout the study period. Responses were collected based on the questionnaire, including: How do you like the taste? How do you like the aroma? How do you like the appearance and texture and overall product? One-way ANOVA (regression analysis) and *t*-test with Welch correction, with *p* < 0.05 as significantly different, were used for the evaluation of differences between the gummy and changes over 45 days of storage.

## 4. Results

### 4.1. Preparation and Optimization of Vitamin D Nanoemulsion

**Figure 2:**
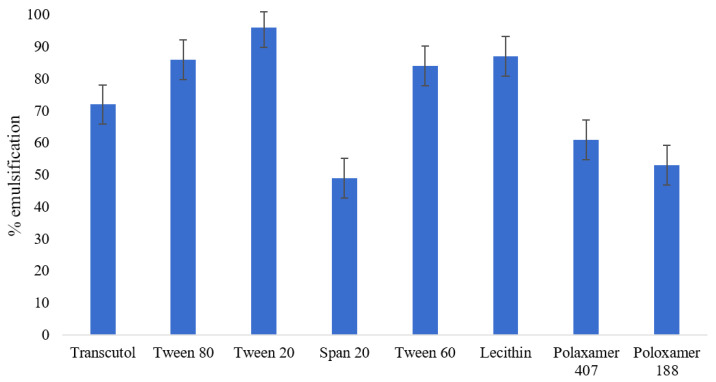
Comparative corn oil emulsification potential of various screened surfactants.

Based on the previously reported solubility of vitamin D, corn oil was selected as the oily phase. Results of the emulsification study (Figure 2) showed that tween 20 has maximum potential to emulsify corn oil followed by lecithin. The decreasing order of emulsification potential among was as follows: tween 20 (96%) > lecithin (87%) > tween 80 (84%) > tween 60 (84%) > transcutol (72%) > poloxamer 407 (61%) > poloxamer 188 (53%) > span 20 (49%). From the results obtained, tween 20 and lecithin were selected as surfactant and co-surfactant, respectively, for the preparation of the vitamin D nanoemulsion. 

**Figure 3:**
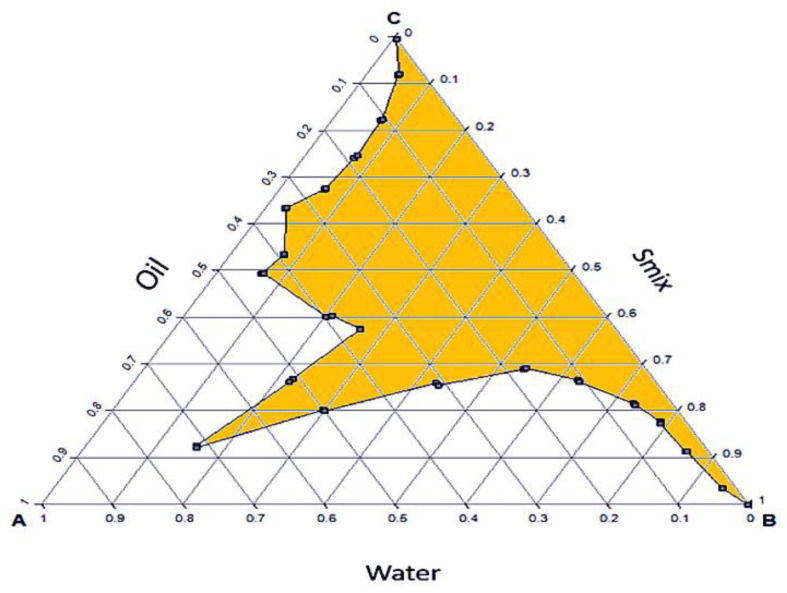
Pseudo-ternary Phase diagram of optimized nano-emulsion with corn oil, Smix (tween 20 + lecithin; 2:1) and water.

Data obtained from a grading system based on the visual screening, appearance, and emulsification capacity of formulations prepared using different ratios of Smix and Oil were obtained and are tabulated in Table 1. Data revealed that Smix ratio 2:1 resulted in a clear and stable nanoemulsion. Thus, Smix 2:1 was selected for pseudo ternary analysis to obtain the optimized formulation. The nanoemulsion region is shown by a colored region in the pseudo-ternary phase diagram (Figure 3). From the results, it was evident that 3% corn oil, 3% tween 20 and lecithin in a ratio of 2:1 and 94% water resulted in the optimized vitamin D nanoemulsion utilized for further evaluation. 

### 4.2. Characterization of Vitamin D Nanoemulsion

**Figure 4:**
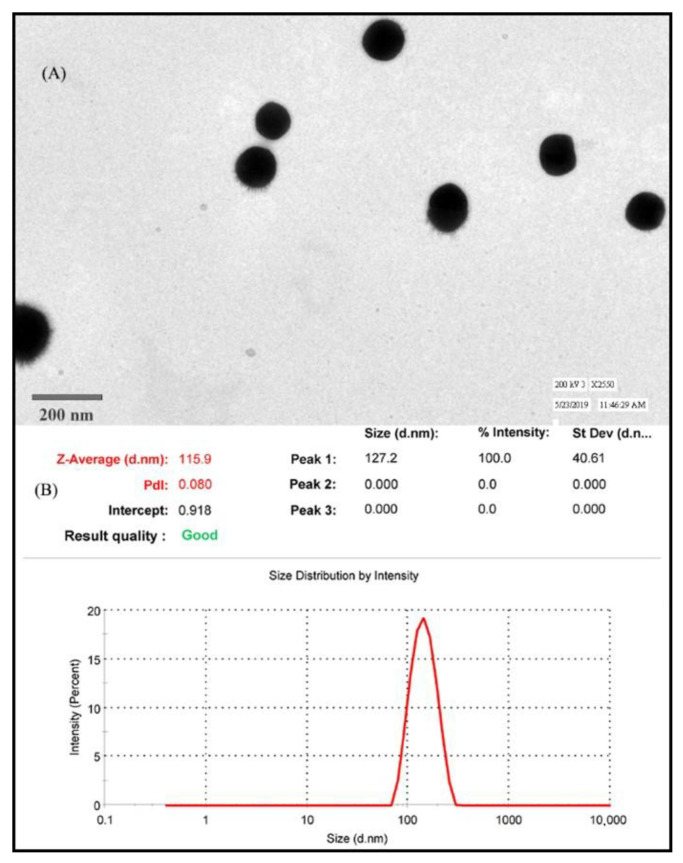
(**A**) HI-RES TEM micrograph of NE. (**B**) Particle size distribution of optimized NE.

As illustrated in Figure 4B, the average particle size and particle size distribution of the optimized vitamin D nanoemulsion were found to be 118.6 ± 4.3 nm and 0.11 ± 0.30, respectively. The zeta-potential was found to be −27 ± 0.82 mV, indicating that the formed nanoemulsion was stable.

TEM revealed that the vitamin D nanoemulsion was spherical (Figure 4A). The droplet size measured by TEM (127 ± 7.2 nm) was slightly larger, but not significantly different from that measured by dynamic light scattering (118.6 ± 4.3 nm). The slightly larger size observed in TEM could be due to the measurement of the hydration layer surrounding the globule, which was not measured using DLS due to difference in principle.

### 4.3. Stability Study of Vitamin D Nanoemulsion

**Figure 5:**
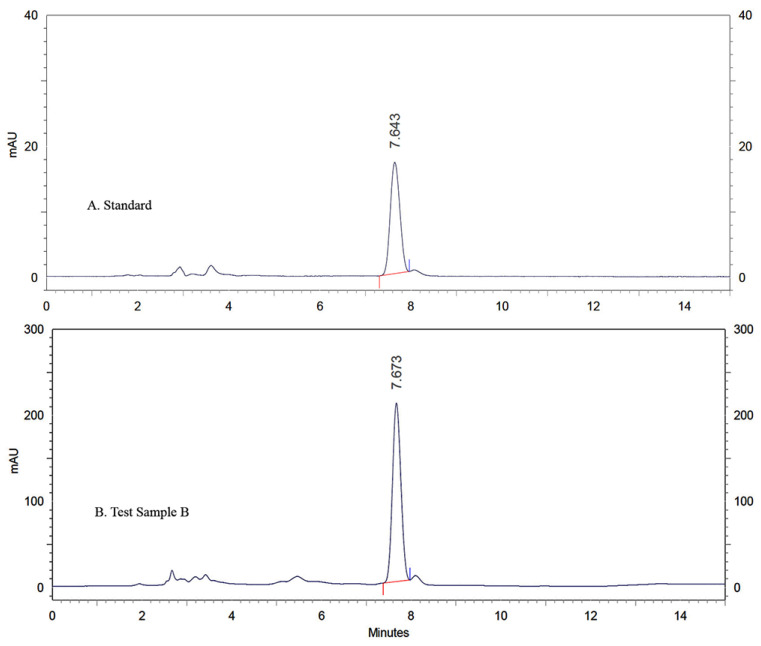
HPLC Chromatograph of (**A**) Standard solution (**B**) Test sample B.

A representative chromatogram of vitamin D is shown in Figure 5A,B. The standard solution of a known concentration of vitamin D (1 μg/mL) was analyzed five times for robustness. The data obtained for the regression plot of vitamin D by HPLC are shown in Table 3.

The linear equation for the calibration plot was used to quantify the concentration of vitamin D on days 0, 15, and 30. Vitamin D in corn oil solution degraded by 5.49% in 15 days and 8.97% in 30 days, whereas the nanoemulsion formulation degraded by 2.94% and 7.63% on day 15 and day 30, respectively (Table 4).

### 4.4. Texture Analysis

**Figure 6:**
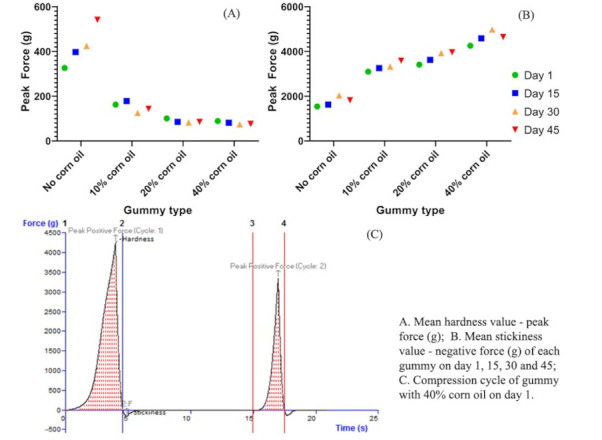
Graphical representation of (**A**) peak hardness cum (**B**) peak stickiness of each gummy on day 1, 15, 30 and 45 and (**C**) force-time curve of 40% corn oil gummy on day 1.

The gummies prepared using different concentrations of corn oil as plasticizer were subjected to texture analysis. The maximum force during the initial compression was used to determine hardness (g) and the negative force with which the probe disconnects from the sample over its way up after compressing was termed as stickiness (g). The mean hardness values and mean stickiness values of each gummy type on day 1, 15, 30, and 45 are shown in Table 5. Gummies without corn oil were softest on day 1 (1540 ± 125 g) but they were extremely soft and difficult to handle and not in the range of an acceptable marketed gummy. Although gummies with 40% corn oil had the lowest stickiness value of the four (76 ± 8 gm on day 45) due the excess of oil present, they were the hardest. As shown in Figure 6, gummies with 10% corn oil had the optimum hardness value (3100 ± 200 g on day 1 and 3600 ± 210 g on day 45), and stickiness was also minimal. It was noted that the gummies prepared with 10% corn oil had excellent hardness and texture up to 30 days and this was retained up to 45 days on further storage.

### 4.5. Sensory Analysis

The Placebo, Nano, and Marketed gummies were compared and the data obtained via sensory evaluation conducted at Day 1 and at Day 45 following storage are presented in Table 6. The highest score for taste was observed for the Marketed gummy. However, the difference between the score compared to placebo and nano gummy was insignificant (*p* > 0.05). Further, storage for a period of 45 days had no detrimental effect on the taste and the scores for all the gummies remained same. The aroma of Placebo and Nano gummies was significantly better than the Marketed gummy (*p* = 0.01; *p* < 0.05). Following 45 days storage, the loss of aroma was observed for all the gummies, but the loss was maximum for the Marketed gummy. Although the appearance and texture of the Marketed gummy was better than Nano gummy (*p* = 0.029; *p* < 0.05), the nano gummy retained both texture and appearance without any significant change (*p* = 0.45) over 45 days of storage. Overall liking of the Nano gummy was comparable to Marketed gummy and was similar on day 1 and day 45 without any significant difference (*p* = 0.4).

## 5. Discussion

The present study explores the potential of a novel nano matrix-based gummy (Nano gummy) to deliver vitamin D. The novel matrix not only prevented the degradation of vitamin D, but also enhances the compliance. Our results suggested that vitamin D was well preserved in O/W nanoemulsion and remained stable over storage. Further, sensory analysis studies suggested that palatability and consumer acceptability were well achieved.

The Nano gummy described in the present work was prepared by a two-step procedure. Firstly, vitamin D loaded nanoemulsions were formed using spontaneous emulsification and aqueous titration procedure. Based on the solubility studies and published literature, corn oil was selected as oil phase. A previous study supported that the in vitro digestibility and bioaccessibility of vitamin D were maximum in long chain triglycerides such as corn oil compared to medium chain triglycerides [34,35]. Studies conducted by Yang et al. [36], Qian et al. [37], and Rao et al. [38] showed similar results where long chain triacylglycerols, viz. corn oil, displayed better bioavailability characteristics of fat-soluble vitamins in contrast to indigestible oils due to the improved solubilization ability of mixed micelles produced by long chain fatty acids [39]. From the results of the emulsification study, tween 20 and lecithin with maximum potential to emulsify corn oil (96% and 87%, respectively) were selected as surfactant and co-surfactant, respectively.

Pseudo-ternary phase diagrams were drawn to locate the nanoemulsion zones, where oil, surfactant, co-surfactant, and water exist as clear and homogeneous dispersions. Ternary phase diagrams provide information on the best combinations to use for the preparation of nanoemulsion. The droplet size of the nanoemulsion is critical as it influences the solubility as well as bioavailability and it is well established that smaller size droplets result in enhanced solubility and bioavailability owing to the higher surface area [34,35].

The phase analysis demonstrated that when the surfactant: co-surfactant ratio was 2:1, the largest proportion of oil was integrated in the nanoemulsion system and smaller size nanoemulsions. The 3% corn oil, 2% tween 20, 1% lecithin, and 94% water resulted in an optimized vitamin D nanoemulsion with globule size 118.6 ± 4.3 nm and particle size distribution of 0.11 ± 0.30. The zeta potential of nanoemulsion is important because its value can be linked to colloidal dispersion stability. In a dispersion, the zeta potential demonstrates the extent of repulsion between contiguous, identically charged particles. A higher zeta potential indicates stability for particles and molecules of sufficiently small size. In cases of lower zeta potential, repulsive forces are weaker compared to attractive forces, leading to the breaking and flocculation of dispersions. Colloids having a large zeta potential (either negative or positive) are deemed electrically stable [48,49]. The optimized formulation displayed zeta-potential of −27 ± 0.82 mV, indicating that the developed nanoemulsions are stable. Using TEM, the structural and dimensional characterization of the optimized vitamin D nanoemulsion was accomplished. The nanoemulsion droplet structure was confirmed to be spherical, and each droplet had a discrete region surrounding it, which suggested vitamin D encapsulation. The size of the nanoemulsion droplets obtained by TEM was in agreement with that obtained by DLS. Blackness in the core of particles as observed in TEM image could be attributed to the solubilization of vitamin D in the oil.

During the stability study, the results showed that vitamin D nanoemulsion retained the vitamin D concentration quite well, although it was exposed to water in the emulsion. Jiang et al. [23] showcased similar behavior when encapsulating vitamin D in carrier oils and storing away from light, which prevents its otherwise easy degradation. This study confirmed the stability of vitamin D in the nanoemulsion upon storage for extended time periods. The vitamin D degradation was only 7.63% in nanoemulsion compared to 8.97% in oil following 30-day storage. The stability results do not appear to be distinctly different but are significant because in the nanoemulsion, the vitamin D, prone to hydrolysis, was in contact with water.

In the second step, the optimized nanoemulsion was mixed with hydrocolloid matrix to form the Nano gummy. It was hypothesized that the Nano gummy might improve the bioavailability of vitamin D, although this was not tested in the present research. The improved bioavailability of vitamin D from Nano gummy could be explained by two mechanisms. Firstly, gummies are meant for chewing in the mouth where they are mixed with saliva and begin to dissolve. Most likely, the released nanoemulsion from the Nano gummy in the buccal cavity will allow enhanced permeation of vitamin D and thus improved bioavailability. Such enhancement of buccal permeation by nanoemulsions has been previously reported [39,50,51]. Secondly, the released nanoemulsion from the Nano gummy will disperse in the gastric fluid. The nanoemulsion results in the enhanced solubilization of vitamin D and its subsequent absorption from GIT, resulting in improved bioavailability. Thus, the Nano gummy could significantly enhance the bioavailability of vitamin D encapsulated in nanosized emulsion droplets in hydrocolloid matrix [50].

While designing gummies, oil incorporation played an important in the texture properties of gummies. Gummies with corn oil 10% exhibit optimum hardness value throughout the study. The addition of oil led to a steep increase in hardness value, but for gummies the optimum hardness value ranges around 3100 g force. After 3100 g force, the gummies become harder and firmer. Texture profile analysis may further be used to track changes in texture during the drying process of gummy confections. The stickiness results showed that the gummies with the lowest content of oil, i.e., 10% oil, exhibited more adhesiveness throughout the study, as can be observed in Table 5. The stickiness value increases with a decrease in oil content. Stickiness for gummies with 40% oil content was even lower, but they became hard and deteriorated the chewy feel. The texture of the gummies is also affected by their age, because the water content changes over time and water plays an essential element in maintaining texture [52]. Concentrations of other additives were kept constant throughout the experiment. Sucralose is an artificial sweetener, while sorbitol and xylitol are sugar alcohols. These act as sweeteners and impart that likeliness among children to willingly consume the product. Various flavors improve the aroma and overall taste of the product since these factors play an important role in overall liking of the product. Homogenization speeds were limited to 1000 rpm as higher homogenization speeds may lead to instability or breaking of the incorporated emulsion.

Although texture can be measured using both instrumental and sensory techniques, it has been demonstrated that sensory analysis when coupled with instrumental analysis yields better attributes [53] for a consumer-oriented product. The sensory assessment technique is widely used in food product development, its quality control, monitoring of shelf life, consumer acceptance, and some experts suggest it to be one of the most sensitive and reliable approach in certain situations [54]. In the current study, the formulated gummy retained its texture and overall acceptance over 45 days of storage at room temperature. This signifies a stable product in terms of consumer perception. Unvarying taste over storage indicated that excipients were inter compatible and incorporated nano-emulsion jacketed vitamin D quite well. Although the formulated confectionary lost significant aroma on storage based on the sensory scores, this could be improved by modifying flavoring agents.

## 6. Conclusions

The nanoemulsion-based matrix system using edible oil, emulsifier and hydrocolloid proved to be an effective approach to develop stable, nano enhanced vitamin D gummies that are readily consumer acceptable. The developed Nano gummy could enhance the vitamin D bioavailability. However, prolonged stability studies and gummy evaluation are required before scale up.

## Figures and Tables

**Table 1:** (**A**). Grading system of oil and Smix based on appearance and emulsification time. (**B**). Visual screening data of nano emulsions using different ratio of Smix and oil.

A. Grade	Parameter			Self-Emulsion Time (min)	
1.	Rapid, clear nanoemulsion			<1	
2.	Rapid, slight hazy nanoemulsion			<2	
3.	Slow, turbid emulsion			>3	
4.	No emulsification			>4	
**B. Grading based on appearance**					
*Ratio of Smix: Oil*	*Co-surfactant to surfactant ratio*				
	1:1	2:1	1:2	1:3	3:1
10:3	1	1	1	1	1
09:3	1	1	1	1	1
08:3	2	1	1	1	1
07:3	2	1	1	2	1
06:3	2	1	2	2	1
05:3	3	1	2	2	2
04:3	3	1	2	3	2
03:3	4	1	2	4	2
02:3	4	2	3	4	3
01:3	4	4	4	4	4

**Table 2:** Nano gummy formula with 10 wt. % corn oil.

Ingredients	Amount (wt. %)
O/W nanoemulsion	34.29
Gelatin	10.29
Sorbitol	13.20
Xylitol	30.79
Sucralose	0.38
Citric acid	0.38
Lime flavor	0.66
Corn Oil	10.00

**Table 3:** Average retention area, retention time and peak height of stock solution.

Concentration (µg/mL)	Average Area	Retention Time	Height	Area %
1	4,132,567	7.643	284,993	100
1	4,134,052	7.620	285,379	100
1	4,142,904	7.660	286,712	100
1	4,130,736	7.663	284,132	100
1	4,140,783	7.677	285,363	100
Mean	4,136,208.4	7.652	285,315.8	100
Std. Dev.	5329.52	0.022	930.21	NA

**Table 4:** Concentration reduction and percent reduction of vitamin D on day 15 and 30.

Test Day	Sample A (IU/mL)	% Reduction	Sample B (IU/mL)	% Reduction
Day 0	289,302.01	0	38,282.13	0
Day 15	273,420.01	5.49	37,155.68	2.94
Day 30	263,360.20	8.97	35,360.46	7.63

**Table 5:** (**A**) Mean hardness value (Peak force (g) mean ± STDV) and (**B**) mean stickiness value (negative force (g) mean ± STDV) of each gummy on day 1, 15, 30 and 45.

A. Mean Hardness Value (Peak Force (g) Mean ± STDV) of Each Gummy				
Oil Content in Gummy	Day 1	Day 15	Day 30	Day 45
No corn oil	1540 ± 125	1620 ± 100	2030 ± 150	1820 ± 155
10% corn oil	3100 ± 200	3260 ± 155	3330 ± 165	3600 ± 210
20% corn oil	3410 ± 265	3630 ± 230	3930 ± 195	3970 ± 265
40% corn oil	4260 ± 165	4600 ± 185	5000 ± 205	4650 ± 195
**B. Mean Stickiness Value (Negative Force (g) Mean ± STDV) of Each Gummy**				
No corn oil	325 ± 45	400 ± 35	425 ± 60	550 ± 60
10% corn oil	160 ± 20	180 ± 30	125 ± 25	145 ± 33
20% corn oil	100 ± 15	85 ± 15	80 ± 7	85 ± 12
40% corn oil	90 ± 14	80 ± 14	75 ± 9	76 ± 8

**Table 6:** Average scores from the sensory evaluation on Day 1 and day 45.

Day 1	Placebo Gummy	Nano Gummy	Marketed Gummy
Taste	7.1 ± 1.7	6.7 ± 1.6	7.3 ± 1.4
Aroma	7.4 ± 1.3	7.0 ± 0.8	5.4 ± 0.7
Appearance/texture	7.0 ± 0.6	6.8 ± 0.9	7.6 ± 1.2
Overall liking	6.9 ± 0.9	6.6 ± 0.8	7.2 ± 1.0
**Day 45**			
Taste	7.2 ± 1.4	6.6 ± 1.0	7.4 ± 0.7
Aroma	6.7 ± 0.7	6.4 ± 1.1	4.5 ± 0.6
Appearance/texture	7.1 ± 0.8	6.9 ± 1.2	7.4 ± 0.9
Overall liking	6.7 ± 0.6	6.5 ± 0.6	7.3 ± 1.1

## Data Availability

This study did not report any data (All the data is included in the current manuscript).
